# L-Type Amino Acid Transporter 1-Utilizing Prodrugs of Ketoprofen Can Efficiently Reduce Brain Prostaglandin Levels

**DOI:** 10.3390/pharmaceutics12040344

**Published:** 2020-04-11

**Authors:** Ahmed Montaser, Marko Lehtonen, Mikko Gynther, Kristiina M. Huttunen

**Affiliations:** School of Pharmacy, Faculty of Health Sciences, University of Eastern Finland, P.O. Box 1627, FI-70211 Kuopio, Finland

**Keywords:** membrane transport, transporters, neuro-inflammation, LAT1-utilizing pro-drugs, prostaglandin E2, targeted brain delivery, blood-brain barrier, COX peroxidase assay

## Abstract

In order to efficiently combat neuroinflammation, it is essential to deliver the anti-inflammatory drugs to their target sites in the brain. Pro-drugs utilizing the L-type amino acid transporter 1 (LAT1) can be transported across the blood-brain barrier (BBB) and the cellular barriers of the brain’s parenchymal cells. In this study, we evaluated, for the first time, the efficacy of LAT1-utilizing prodrugs of ketoprofen (KPF) on cyclooxygenase (COX) enzymes in vitro and prostaglandin E2 production in vivo by using an enzymatic assay and liquid chromatography- tandem mass spectrometry method, respectively. Aliphatic amino acid-conjugated pro-drugs inhibited the peroxidase activity of COX in vitro in their intact form (85% inhibition, IC50 ≈ 1.1 µM and 79%, IC50 ≈ 2.3 µM), which was comparable to KPF (90%, IC50 ≈ 0.9). Thus, these compounds acted more as KPF derivatives rather than pro-drugs. In turn, aromatic amino acid-conjugated pro-drugs behaved differently. The ester pro-drug inhibited the COX peroxidase activity in vitro (90%, IC50 ≈ 0.6 µM) due to its bioconversion to KPF, whereas the amide pro-drug was inactive toward COX enzymes in vitro. However, the amide pro-drug released KPF in the mouse brain in sufficient and effective amounts measured as reduced PGE2 levels.

## 1. Introduction

Inflammation is an adaptive cellular response intended to counter-act abnormal stimuli [1]. These stimuli can be exogenous, e.g., exposure to foreign triggers (i.e., infections) or endogenous due to homeostatic imbalance in physiological processes such as when it occurs in autoimmune diseases. The inflammatory process is an autonomous and beneficial physiological response aimed to repair the malfunctioned cells or protect tissue. However, if inflammation persists and fails to resolve the inflammatory trigger, it can turn into a pathological situation such as encountered in arthritis and neurodegenerative diseases. 

It is widely acknowledged that neuroinflammation is a prominent hallmark encountered in almost all neurological diseases [2]. However, it is still not clear whether neuroinflammation is the main cause or a symptom of the disease. One common pathway of inflammation is initiated by two enzyme isoforms called cyclooxygenases 1 and 2 (COX1 and 2) [3]. COX1 is a constitutive enzyme that is expressed in most tissues under normal conditions. COX 2 is an inducible form expressed mainly in response to pathological stimulus (i.e., inflammation). COX enzymes catalyze the conversion of arachidonic acid in a multi-step process to several inflammatory mediators such as prostaglandins (PGs) and thromboxane (T). First, arachidonic acid is generated by the action of phospholipase A2 on fatty acids. Thereafter, COX enzymes initiate bis-dioxygenation of arachidonic acid to cyclopentane hydroperoxy endoperoxide (PGG2) followed by a reduction reaction step by peroxidase activity of COX enzymes to generate the corresponding alcohol (PGH2) [4,5]. In turn, PGH2 is converted to prostanoids (PGs and thromboxanes) by the action of tissue-specific isomerases. Nonsteroidal anti-inflammatory drugs (NSAIDs) inhibit COXs and decrease the production of PGs, and, thereby, alleviate pain and decrease inflammation. Ketoprofen (KPF) is an example of a COX inhibitor that mainly acts peripherally since it poorly penetrates through the blood-brain barrier (BBB) [6]. Similarly, the brain uptake of other NSAIDs is limited due to the BBB and the plasma protein binding [7]. Hence, there is a huge need to develop anti-inflammatory drugs that can pass through the BBB and reach their target sites in the brain parenchymal cells in a sufficient and effective amount. Alternatively, the brain uptake of NSAIDs can be improved via the carrier-mediated transport [8].

Transporter-mediated brain uptake is considered one of the most promising ways to deliver small molecular weight drugs into the brain [9]. For instance, L-type amino acid transporter 1 (LAT1) is involved in the brain delivery of some FDA-approved and clinically used drugs such as levodopa, baclofen, melphalan, and gabapentin [10]. LAT1 is highly expressed in blood brain barrier (BBB) in comparison to other healthy tissues [11]. Moreover, this transporter is highly expressed in both the luminal and abluminal sides of the BBB [12] as well as in the brain parenchymal cells such as astrocytes, microglia, and neurons [13]. LAT1-utilization has not only been able to enable the brain delivery of ferulic acid, dopamine, valproic acid, and KPF prodrugs [14,15,16,17], but also to increase the extent of uptake in brain parenchymal cells in vitro [13]. Unlike other transporters expressed at the BBB, the function and protein expression of LAT1 is not changed in Alzheimer’s disease (AD)-like or Parkinson’s disease (PD)-like pathology [18,19]. Therefore, LAT1-utilization is considered a promising transport method for intra-brain targeted drug delivery. 

**Figure 1:**
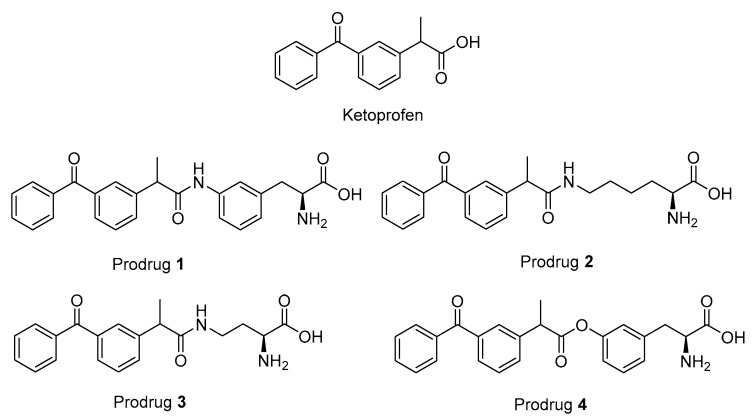
Structures of ketoprofen (KPF) and the studied pro-drugs 1–4.

There is increasing evidence that chronic use of some NSAIDs can exert protective effects against neurodegenerative diseases such as Alzheimer’s disease [20,21,22]. However, often the long-term studies have to be interrupted due to the peripheral adverse effects of NSAIDs. This, in turn, have complicated drawing a plausible conclusion about this approach. These adverse effects arise mainly due to the inhibition of the constitutive COX1 in intestines that leads to gastrointestinal erosions and, in liver and kidneys, it leads to hepatic and renal insufficiency [23,24,25]. Therefore, a more COX2-selective class of NSAIDs have been developed, but, unfortunately, their long-term use has not been favored due to their cardiovascular complications [26]. Alternatively, targeting the NSAIDs into the brain via a pro-drug approach seemed to be a promising solution [8]. Previously, we have reported a brain-targeted prodrug of KPF (PD1 in Figure 1) that utilizes LAT1 for its brain uptake across the BBB and its intracellular localization within the brain parenchymal cells [13,14]. Moreover, this pro-drug releases KPF into the brain and liver with a ratio of 0.5 and 0.1, respectively, as compared to the KPF treatment and with only a minor release of KPF in plasma [14]. The brain partition coefficient value (K_p,u, brain_ = AUC_u, brain_/AUC_u, plasma_, where the unbound fraction was measured with equilibrium dialysis) was 0.13 for the KPF released from the pro-drug while the corresponding value for KPF itself was 0.01. Therefore, it would be possible to examine the long-term efficacy of these kinds of pro-drugs since they would have minimal peripheral exposure and possible higher intracellular disposition. In this study, we investigated the properties of four selected pro-drugs conjugated with an aliphatic or aromatic amino acid via an ester or amide bonds to KPF (LAT1-utilizing pro-drugs) on COX peroxidase activity in vitro. Furthermore, one pro-drug was selected to evaluate its inhibitory effect on acute and subacute lipopolysaccharide (LPS) -induced prostaglandin E2 (PGE2) production in mice brains.

## 2. Materials and Methods

### 2.1. Studied Compounds

The studied LAT1-utilizing pro-drugs of KPF (1–4 in Figure 1) have been previously designed according to the 3-Dimensional Quantitative Structure Activity (3D-QSAR) model of the rat Lat1 binding site and, therefore, the syntheses of these compounds have been published elsewhere [14,27]. In these studies, pro-drugs 1–3 were found to be stable in Tris-buffered saline (TBS) pH 7.4 and 0.1 M NaOH as well as in plasma and a S9 subcellular fraction of the liver and brain. In comparison, pro-drug 4 (PD4) was found to release KPF quantitatively in human, mouse, and rat plasma (half-lives ranging from 6 to 30 min), human and rodent liver S9 subcellular fraction (half-lives ranging from 13 to 170 min), and the rodent brain S9 subcellular fraction (half-lives ranging from 52 to 74 min) and was completely bio-converted in 0.1 M NaOH within 30 min, while being stable in TBS-buffer at a pH of 7.4. Although pro-drugs 1–3 were stable in vitro, all of the pro-drugs released KPF in vivo in the plasma and liver. Furthermore, the amide pro-drug 1 (PD1) was the only pro-drug able to release KPF in the brain.

### 2.2. Cell-Based Fluorometric Assay of COX Peroxidase Activity

Immortalized mouse microglia, BV2 cells (passage 12), were cultured in RPMI-1640 medium with L-Gln and sodium bicarbonate (Sigma-Aldrich, Co, St. Louis, MO, USA) and supplemented with 100 U/ml of penicillin and streptomycin (EuroClone, Milan, Italy). A total of 8 × 10^6^ cells per culture plate were lysed with 1.5 ml of the commercial lysis buffers containing protease inhibitors and DNAse (Abcam©, ab193970 and ab193971) on top of ice. The cells were scraped off the culture plate and kept on ice for 20 min while vortexing every 5 min. Thereafter, the cell lysates were centrifuged at 18,000× *g* at 4 °C for 5 min and then the supernatants were transferred to clean Eppendorf tubes. 

In 96-well plates, 10 µl of dimethyl sulfoxide (DMSO), KPF or pro-drugs dissolved in dimethyl sulfoxide (DMSO) were pipetted in a triplicate manner to final concentrations of (0.5–40) µM. A total of 20 µl of the BV2 cell lysates was pipetted and the well plate was kept on ice during the experiment. A 50-µl volume of the reaction mixture containing 100 µM of 10-acetyl-3,7-dihydroxyphenoxazine (Cayman Chemical, Co, Ann Arbor, MI, USA) and 80 nM of hemin (99%, porcine, ACROS Organics™, Fischer scientific Co, Ann Arbor, MI, USA) diluted in 100 mM sodium phosphate buffer at a pH of 7.4 were added to each well. The reaction was initiated by adding 20 µl of arachidonic acid micelles prepared by diluting 200 mM arachidonic acid in methanol (Density 0,922 g/ml, Nu-Chek-Prep, Elysian, MN, USA) with 0.1 M sodium hydroxide (1:1) and with further dilution with deionized water to a final concentration of 2 mM. Two sets of wells were utilized for sample and arachidonic acid blanks. The fluorescence was measured in a 30-min endpoint mode by the Envision plate reader (EnVision, Perkin Elmer, Waltham, MA, USA) at λ_ex_ 535 nm and λ_em_ 587 nm. The IC50 values (the concentration of tested compound inhibiting enzyme activity by 50%) were calculated using the concentration-response curve (0.5–40 µM) by GraphPad Prism v. 5.03 software (GraphPad Software, San Diego, CA, USA). 

### 2.3. Study Design and Animals

The animal experiments were conducted according to the Council of Europe (Directive 86/609) and Guide for the Care and Use of Laboratory Animals. The animal procedures were approved by the Finnish National Animal Experimental board (ESAVI-2015-003347). All animals were adult male C57BL/6JOlaHsd mice (Jackson Laboratories, Bar Harbor, ME, USA) supplied by Envigo (Venray, Netherlands). The neuroinflammation was induced by treatment with lipopolysaccharides (LPS) from Escherichia coli O55:B5 (Sigma-Aldrich, Co) (250 µg/kg i.p. once per day for 3 consequent days) after which the animals were killed by decapitation on the fourth day. The animals were allocated to one of four treatment groups (*n* = 6 per group). The anti-inflammatory efficacy of KPF and PD1 was investigated by injecting the mice either simultaneously with LPS administration (on days 1, 2 and 3) and, on the fourth day, to evaluate the preventive effect (KPF/PD1 plus LPS) or after the LPS had already triggered inflammatory effects on the third and fourth days (KPF/PD1 after LPS). PD1 or KPF (25 µmol/kg) was given according to the above-mentioned regimen as i.p. injection. The control group of LPS-treated mice was injected with 0.9% NaCl solution i.p once per day for 3 days, which was followed by decapitation on the fourth day. The following standard laboratory conditions for animal housing were used: 12/12 h light-dark day cycle, food pellets (Lactamin R36, Lactamin AB, Södertälje, Sweden) and tap water consumption ad libitum. After decapitation, mouse blood and brain samples were collected. The plasma was separated by centrifugation at 1500× *g* for 6 min. The plasma layer was centrifuged again at 12,000× *g* to remove the platelets. Plasma was stored at −80 °C until analysis. The brains were snap-frozen in liquid nitrogen and stored at −80 °C until analysis.

### 2.4. Prostaglandin E2 (PGE2) Quantification

The weighed frozen brain samples (approximately 20 mg) were transferred into Eppendorf tubes. Subsequently, 80% (*v/v*) aqueous solution of a prechilled methanol (HPLC grade) was added with the adjusted volume based on tissue weight (400 μL of solvent per 20 mg of tissue). The tissue was ground with a small pestle grinder on dry ice. Two cycles of centrifugation 14,000× *g* for 10 min at 4 °C were applied. The supernatant was filtered through a 0.2-μm syringe filter (Acrodisc® CR 13 mm Syringe Filter, PALL life science, Ann Arbor, MI, USA), which was followed by LC-MS/MS analysis described previously [28]. Agilent 1200 Series Rapid Resolution LC System with Agilent 6410 triple quadrupole mass spectrometer was equipped with an electrospray ionization source (Agilent Technologies Inc., Wilmington, DE, USA). The Poroshell 120 EC-C-18 column (50 mm × 2.1 mm, 2.7 μm) was used for liquid chromatography prior to mass spectrometric analysis. The eluents were water containing 0.1% (*v/v*) formic acid (eluent A) and acetonitrile (eluent B). An isocratic gradient with 10% eluent B was applied. The flow rate was 0.2 mL/min, the column temperature was 40 °C, and the injection volume was 5 μL. The following mass spectrometry parameters were used: negative ion mode for drying gas (nitrogen) temperature of 300 °C, drying gas flow rate at 8 L/min, nebulizer pressure of 20 psi, and capillary voltage of 3500 V. The fragmentor voltages were 180 V and the collision energy was 6 V. Analyte detection was performed using multiple reaction monitoring with the transitions 351.4 → 315.4 and 351.4 → 271.1 (qualifier). The data was acquired using the Agilent Mass Hunter Workstation Acquisition software (Data Acquisition for Triple Quadrupole Mass Spectrometer, version B.03.01) and processed with Quantitative Analysis (B.04.00) software. The lower limit of quantification (LLOQ) for PGE2 was 1 nM. The linearity of the calibration curve (1–100 nM) as well as the selectivity, accuracy, and precision of the method were acceptable. Within-run accuracy and precision of quality control samples were ±20% of the nominal concentration.

The plasma samples were prepared in a similar manner. Plasma samples were transferred to Eppendorf tubes and 80% (*v/v*) aqueous solution of a pre-chilled methanol was added (1:4, *v/v*). The samples were gently shaked and incubated for 6 h at −80 °C, which was followed by centrifugation at 14000× *g* for 10 min at 4 °C. The supernatant was filtered using a 0.2 μm syringe filter (Acrodisc® CR 13 mm Syringe Filter, PALL life science, Ann Arbor, MI, USA). The samples were immediately analyzed using the LC-MS/MS method described above [28]. The statistical analyses were performed using GraphPad Prism v. 5.03 software (GraphPad Software, San Diego, CA, USA).

### 2.5. Ketoprofen and Pro-Drug 1 Quantification 

The frozen brain samples were dissected into small pieces (approximately 127 mg on average) and transferred to Bead Ruptor bead beating tubes (2 mL Polypropylene screw cap tubes pre-filled with 1.4 mm ceramic beads, Omni International, Inc., Kennesaw, GA, USA). Milli-Q deionized water (Millipore, Milford, MA, USA) was added (1:3, *w/v*) and the samples were homogenized using Omni Bead Ruptor 24 Elite homogenizer coupled with an Omni BR Cryo cooling unit (Kennesaw, GA, USA). Sample handling and homogenization were conducted at a cool temperature (≈ 4 °C). Samples were prepared by pipetting 50 µl of tissue homogenate to 150 µl acetonitrile acidified with 0.1% formic acid (0.1% FA/ACN), and then centrifuged at 18,000× *g* for 10 min at 4 °C. Standards were prepared in the same way by spiking the standard analyte (5 µl) to the control homogenate (45 µl) and 0.1% FA/ACN (150 µl). The supernatants were diluted (1:2) with (0.1% FA/ACN) containing 200 nM of diclofenac as an internal standard prior to analysis by the LC-MS/MS method described previously [14]. PD1, KPF, and diclofenac (internal standard) were analyzed using Agilent 1260 Infinity LC system coupled with an Agilent 6410 triple quadrupole mass spectrometer with an electrospray ionization source in the positive mode (Agilent Technologies, Santa Clara, CA, USA) by following these transitions (417 → 371.2, 417 → 135.1), (255 → 209), and (296 → 250), respectively. The aqueous mobile phase was 0.1% formic acid in water (A), while the organic mobile phase was 0.1% formic acid in acetonitrile (B). The samples were injected (5 µl) and the compounds were separated using Agilant Zorbax SB-c18 rapid resolution HT 2.1 × 50 mm 1.8 Micron 600 Bar column following the gradient of (0–0.5 min) 20% B, (0.–1 min) 20–95% B followed by (1–6.5 min) 95% B. Then the column was allowed to equilibrate (6.6–9 min) in 20% B with a constant flow rate of 0.5 ml/min. The following instrument optimizations were used: 300 °C sheath gas heat, 6.5 L/min drying gas flow, 25 psi nebulizer pressure, and 4000 V capillary voltage. The fragmentor energies were 175, 100, and 100 V, the collision energies were 17, 10, and 10 V for PD1, KPF, and diclofenac, respectively. The calibration curves for PD1 and KPF were linear with a range of 0.25–25 ng/ml and 0.125–12.5 ng/ml, respectively. The statistical analyses were performed using GraphPad Prism v. 5.03 software (GraphPad Software, San Diego, CA, USA).

## 3. Results 

### 3.1. In Vitro COX Peroxidase Activity

**Figure 2:**
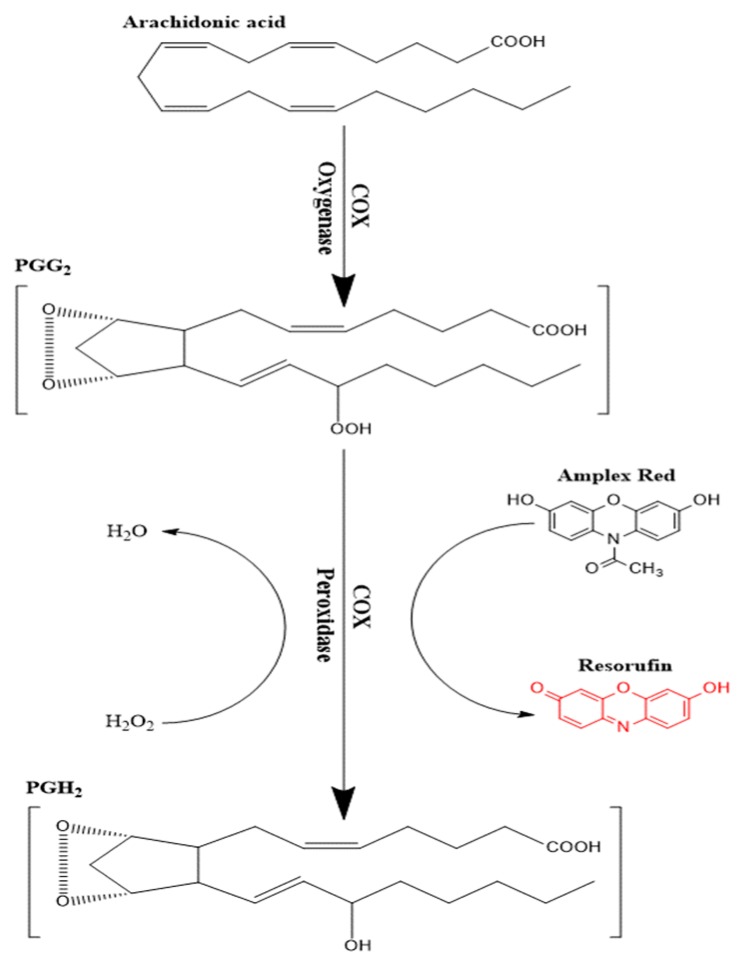
Schematic presentation of the peroxidase activity assay of cyclooxygenase (COX) enzymes.

Enzymatic conversion of a non-fluorescent compound or probe to a fluorescent compound has commonly been utilized in many enzyme assays [29,30,31,32]. Several probes have been utilized following this approach such as scopoletin, homovanillic acid, 4-hydroxyphenyl acetate, and 10-Acetyl-3,7-dihydroxyphenoxazine (Amplex Red©). The latter compound is considered as the most sensitive as it produces resorufin as an end product, which has good stability and low background interferences [33]. Amplex red© is oxidized to resorufin in two successive steps of one-electron transfer in the presence of heme-containing peroxidase enzyme [34]. COX enzymes can specifically couple the reduction of an oxygen acceptor or electron donor such as PGG2 to the oxidation of Amplex red (Figure 2) in the presence of hemin as a co-factor. The end product of Amplex red oxidation is resorufin, which was measured fluorometrically at λex 535 nm and λem 587 nm. By using the parent compound KPF as a reference, the inhibitory effect of the LAT1-utilizing pro-drugs was evaluated against the peroxidase activity.

**Figure 3:**
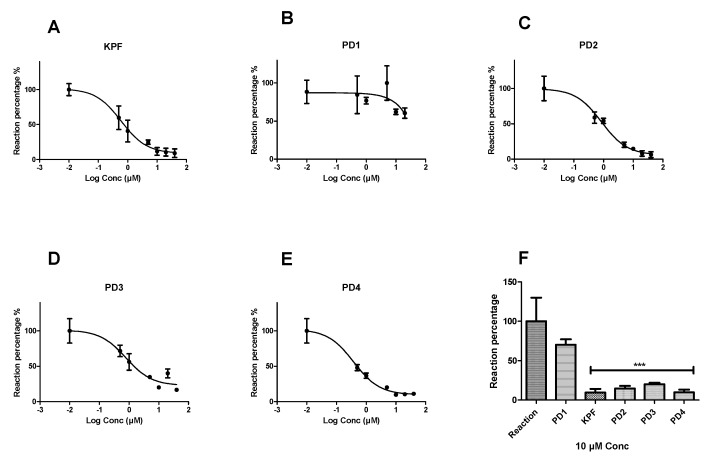
Dose-response curves showing the effect of different concentrations (0.5–40 µM) of the studied compounds on the peroxidase activity of COX. Fluorescence readouts after 30 min incubation time were plotted against the different concentrations of ketoprofen (**A**) PD1, (**B**) PD2, (**C**) PD3, (**D**) and PD4, (**E**) and a comparison of a single-concentration reaction (10 µM) is presented for all compounds (**F**) as a percentage from the reaction wells. Data are presented as mean ± SD (*n* = 3). An asterisk denotes a significant difference from the reaction group (*** *p* < 0.0001, one-way ANOVA, followed by Dunnett’s test).

Peroxidation of arachidonic acid by BV2 cell lysates was inhibited by selective COX inhibitors such as KPF by 90.41 ± 4.6% and IC50 value of 0.85 ± 1.18 µM (Figure 3). Similarly, LAT1-utilizing pro-drugs 2, 3, and 4 (PD2-4) inhibited the peroxidation by (85.32 ± 3.27%, IC50 = 1.05 ± 1.21 µM), (79.89 ± 1.77%, IC50 = 2.26 ± 1.33 µM), and (90.25 ± 3.58%, IC50 = 0.58 ± 1.22 µM), respectively. In contrast, PD1 was not able to inhibit the peroxidation as efficiently as the KPF and the other LAT1-utilizing pro-drugs, even at higher concentrations up to 40 µM.

### 3.2. In Vivo Prostaglandin E2 Production

**Figure 4:**
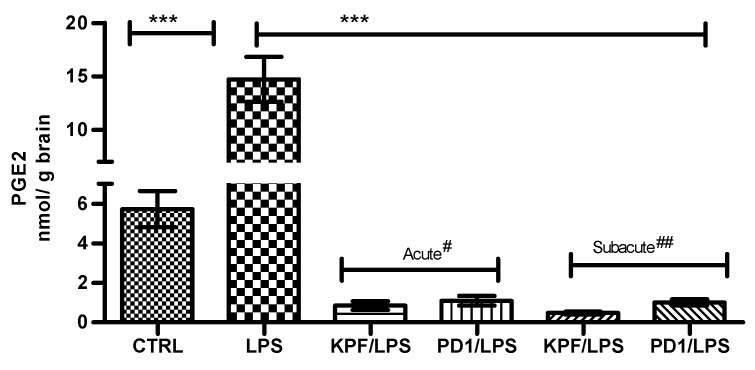
PGE2 amounts in brain tissue samples in control-treated mice, 4 × 250 µg/kg LPS treated mice, 4 × 250 µg/kg LPS treated mice with acute^#^ treatment of 25 µmol/kg PD1 or KPF, and 4 × 250 µg/kg LPS treated mice with subacute^##^ treatment of 25 µmol/kg PD1or KPF. Data presented as mean ± SD, *n* = 6. Asterisks denote a statistically significant difference from the respective control. *** Denotes significance of *p* < 0.0001, one-way ANOVA, followed by Dunnett’s test. ^#^ Acute treatment refers to drug injection on the third and fourth days of LPS treatment. ^##^ Subacute treatment refers to daily drug injection (4 days) simultaneously with the LPS treatment.

PGE2 was quantified in mouse brains of the four treatment groups as well as the control and LPS-induced groups using the LC-MS/MS method described earlier [28]. PGE2 was significantly induced by LPS i.p. injection from 5.72 ± 1.83 to 14.72 ± 4.18 nmol/g of brain tissue (Figure 4). In addition, both KPF and PD1 significantly decreased the LPS-induced PGE2 production in the acute and subacute groups. KPF inhibited the PGE2 levels to 0.86 ± 0.53 and 0.48 ± 0.18, while PD1 inhibited the PGE2 levels to 1.10 ± 0.61 and 1.01 ± 0.41 in the acute and subacute groups, respectively.

**Figure 5:**
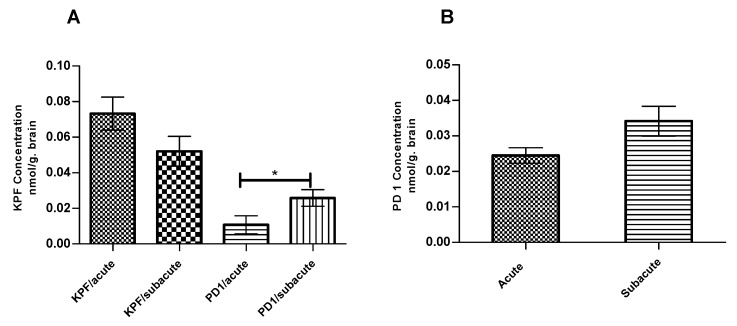
KPF (**A**) Amount in the brain after the i.p administration of 4 × 250 µg/kg LPS and 25 µmol/kg of KPF or PD1 in the acute and subacute groups. PD1 (**B**) amount in the brain after the i.p. administration of 4 × 250 µg/kg LPS and 25 µmol/kg of PD1 in the acute and subacute groups. Data presented as mean ± SD, *n* = 6. * Denotes significance of *p* < 0.05, one-way ANOVA, which is followed by Dunnett’s test.

The amounts of KPF, PD1, and KPF released from PD1 were quantified at the endpoint at which PGE2 levels were measured using the LC-MS/MS described earlier [14]. The total amounts of KPF in the mouse brain were 0.07 ± 0.009 and 0.05 ± 0.008 nmol/g of brain tissue in the acute and subacute groups, respectively (Figure 5A). In contrast, the total amounts of PD1 in the mouse brain were 0.02 ± 0.005 and 0.03 ± 0.01 nmol/ g of brain tissue in the acute and subacute groups, respectively (Figure 5B). However, PD1 was able to release considerable amounts of its parent drug (KPF) in the mouse brain with values of 0.01 ± 0.005 and 0.03 ± 0.005 nmol/g of brain tissue in the acute and subacute groups, respectively.

**Figure 6:**
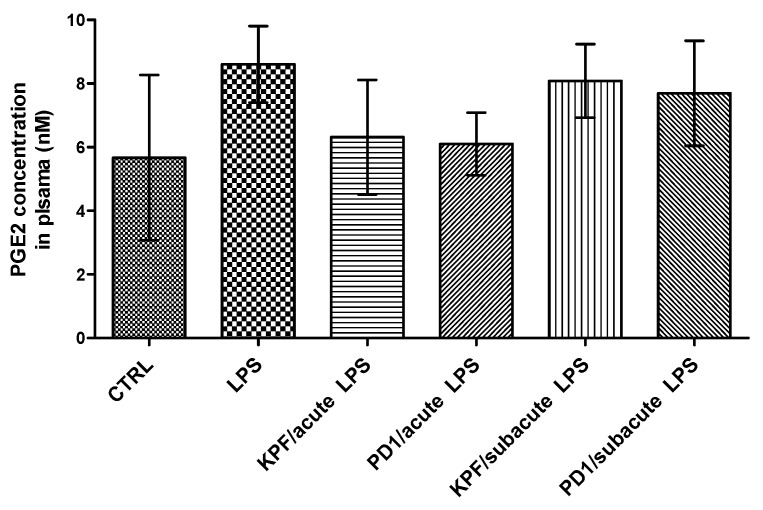
PGE2 concentrations in plasma samples in control-treated mice, 4 × 250 µg/kg LPS treated mice, 4 × 250 µg/kg LPS treated mice with acute treatment of 25 µmol/kg PD1 or KPF, and 4 × 250 µg/kg LPS treated mice with subacute treatment of 25 µmol/kg PD1 or KPF. Data presented as mean ± SD, *n* = 6. Asterisks denote a statistically significant difference from the respective control (*** *p* < 0.001, one-way ANOVA, which was followed by Bonferroni’s test).

The PGE2 levels were also quantified in mouse plasma of the four treatment groups as well as the control and LPS-induced groups by the LC-MS/MS method described earlier [35]. There was no statistically significant difference between the control and LPS-induced groups (Figure 6). Additionally, no significant difference in the amount of PGE2 was found between the four treatment groups and their corresponding control groups.

## 4. Discussion

### 4.1. In Vitro COX Peroxidase Activity

The effect of KPF and the pro-drugs on the COX peroxidase assay was evaluated using the enzymatic fluorometric assay described above (Figure 2). The pro-drugs conjugated with an aliphatic amino acid promoiety (PD2, 3) inhibited COX peroxidase activity in a similar way to KPF (Figure 3). This indicates that these aliphatic pro-drugs are active themselves in vitro without releasing their parent drug as they were stable in the studied medium (see Section 2 and Section 2.1). In contrast, PD1, which is conjugated with an aromatic amino acid promoiety at the meta-position, did not inhibit the peroxidase activity. Since it is known that PD1 was efficiently transported across the BBB via LAT1 after i.p. administration to mice and released KPF within brain parenchyma as reported previously [14,36]. This encouraged us to also evaluate its efficacy in the LPS-induced PGE2 mouse model. Although PD4 is structurally similar to PD1 except that it is an ester pro-drug, it was still able to inhibit peroxidase activity by 90.25 ± 3.58% and the IC50 value of = 0.58 ± 1.22 µM (Figure 3). This could be explained by its rapid bioconversion in the studied medium (see Section 2 and Section 2.1), i.e., there was sufficient KPF released to cause comparable inhibition of the peroxidase activity to KPF. However, since pro-drugs of KPF, which have been conjugated to aliphatic amino acid pro-moieties (PD2 and PD3), were noted to be active toward COX enzyme(s). It would also be interesting to determine the efficacy of those active pro-drugs/derivatives in the future.

### 4.2. In Vivo Prostaglandin E2 Production

Mice treated peripherally with bacterial LPS are shown to display signs of neuroinflammation such as activation of astrocytes and microglia cells as well as the induction of COX2 enzyme [37,38,39,40,41]. A few studies have reported that there is no microglia activation after only a single intraperitoneal injection (i.p.), but the multiple i.p. injections of LPS have caused microglia activation in several studies [41,42]. There is a consensus that the peripherally released inflammatory mediators do not diffuse across the BBB themselves. Instead, they transfer pro-inflammation signals to the brain parenchymal cells. Therefore, repeated injections were utilized in this study to induce COX enzymes and, consequently, induce PGE2 production. 

Because of the repeated i.p. injections of LPS, PGE2 in mouse brain was induced nearly three-fold as compared to the control group (Figure 4). This LPS-induced group acted as a perfect mouse model for neuroinflammation characterized by higher PGE2 values. KPF and PD1 (25 µmol/kg) were administered simultaneously with the repeated LPS dose representing the subacute or prevention group while the administration of compounds at the end of the multiple doses of LPS represented the acute or treatment group. Both KPF and PD1 significantly reduced PGE2 level in the acute and subacute groups and to levels lower than the control group (non-treated) (Figure 4). The PGE2 reduction over the control group was likely attributed to the fact that KPF is a non-selective inhibitor of the constitutive enzyme (COX1) as well as the inducible isoform (COX2).

We also measured the KPF amounts in the mouse brain at the endpoint at which the PGE2 levels were also quantified. Although the amount of released KPF in the pro-drug groups (0.01 ± 0.005 and 0.03 ± 0.005 nmol/g of brain tissue for the acute and subacute groups, respectively) was less when compared to the KPF groups (0.07 ± 0.009 and 0.05 ± 0.008 nmol/g of brain tissue for the acute and subacute groups, respectively) (Figure 5A), the efficacy on PGE2 inhibition remained similar. Thus, the pro-drug released KPF in the brain in lower but, nonetheless, sufficient amounts to elicit the same effects as the parent drug itself. However, the amount of released KPF was quantified in this study from the whole brain tissue and, therefore, we cannot compare how much of KPF was delivered to the target cells, i.e., to activated astrocytes or microglia, in which the target enzymes, COXs, are located. We have previously shown that, although the total brain uptake of PD1 was comparable to KPF itself (K_p, brain/plasma_ 0.0097 and 0.0098, respectively) [36], the LAT1-mediated uptake of PD1 into astrocytes and microglia has been 10–13 and 2–7 times greater, respectively, as compared to KPF [13]. This points to the superiority of LAT1 utilization in transporting the drugs into their intracellular targets and, hence, enhancing their potency and efficacy. Furthermore, as detected in this study, a considerable amount of brain uptake of PD1 had occurred (0.02 ± 0.005 and 0.03 ± 0.01 nmol/ g of brain tissue for the acute and subacute groups, respectively) but it is still detected in its intact form (Figure 5B). In addition, the subacute group, i.e., the mice that had been treated with PD1 for four consecutive days, exhibited a statistically significant increase in the amount of released KPF (0.03 ± 0.005 nmol/g of brain tissue) from PD1 as compared to the acute group, i.e., the mice that received PD1 only for the last two days (0.01 ± 0.005 nmol/ g of brain tissue) (Figure 5A). It is noteworthy that the same difference was not seen with respect to KPF itself. This means that the pro-drug known to be efficiently transported into the brain parenchyma may act as a reservoir and achieve a slow KPF release. Therefore, a full pharmacokinetic study with multiple time points shall be conducted to confirm this finding. Thus, LAT1-utilizing PD1 proved to have a comparable efficacy to KPF, but it may also have a prolonged additional efficacy that can result in improved total effects over a longer-term.

KPF and PD1 did not significantly reduce PGE2 concentrations in plasma in either the acute or subacute treatment groups (Figure 6). Although a minor reduction was seen in the acute groups, this reduction was not statistically significant when compared to the LPS-induced or control groups. This indicates that the effect seen in the brain was completely distinct from the peripheral effects, which is a finding in accordance with the literature [41]. Under these experimental conditions, KPF and PD1 were able to reduce the LPS-induced PGE2 level in the brain, which supports the central efficacy of KPF and its potent LAT1-utilizing pro-drug (PD1). In summary, the LAT1-utilizing pro-drug of KPF (PD1) proofed in this study to be more potent than KPF as well as exhibiting less central and hepatic exposure, which implies less systemic and hepatic side effects as reported earlier [14].

## 5. Conclusions

Ketoprofen conjugated to an aromatic amino acid pro-moiety at the meta-position did not inhibit the COX peroxidase activity in vitro but, after i.p. administration in mice, released the parent drug in the brain. This resulted in sufficient amounts of released KPF and, thus, it exhibited equivalent efficacy as measured via the reduced levels of PGE2 in comparison to the KPF treatment. This study, together with previous findings, shows that the amount of brain uptake is not the only factor influencing effective brain delivery if the final target is located intracellularly. Although the amount of KPF brain uptake was higher than its LAT1-utilizing pro-drug, both compounds displayed equivalent activity against the target site (COX enzyme). It is concluded that this equivalent efficacy was attributed to the higher intracellular brain disposition of the LAT1-utilizing pro-drug as compared to KPF itself. Moreover, in this study, we observed that the repeated dosing with the LAT1-utilizing pro-drug of KPF could achieve a long-term brain exposure to this NSAID, which could be viewed as a form of extended-release. However, this needs to be clarified in the future. The efficacy of the drugs and their brain concentrations could be compared at multiple time points. Therefore, LAT1-utilisation can achieve not only targeted brain delivery but also improved intracellular brain delivery, as we have proven in our earlier studies [13,14,36]. Herein, it demonstrated the efficacy of the pro-drug, as measured by reduction in the concentrations of PGE2, which will permit an investigation of the chronic administration of NSAIDs in the treatment of neurodegenerative diseases while avoiding their peripheral adverse effects.
